# Supplementary material for: Mixed methods approach to examining the implementation experience of a phone-based survey for a SARS-CoV-2 test-negative case-control study in California
Source: PLoS One. 2024 May 21;19(5):e0301070. doi: 10.1371/journal.pone.0301070 (PMC11108220; doi:10.1371/journal.pone.0301070)
Supplement: Attachment — Submitted filename: c4_implementation_response-06mar2024 (jmpry@ucdavis.edu 2).docx [file pone.0301070.s002.docx]

Jake M. Pry, PhD, MPH

Assistant Professor

jmpry@ucdavis.edu | +260 96 3088750 | +1-936-661-6885

Dr. Moustaq Karim Khan Rony

Associate Editor

PLOS One

6 March 2024

Dear Dr. Karim Khan Rony,

Many thanks for re-reviewing our revised manuscript (PONE-D-23-40982-R1), attached, entitled: “Mixed methods approach to examining the implementation experience of a phone-based health research survey investigating risk factors for SARS-CoV-2 infection in California”.

On behalf of my colleagues, I would like to thank you and the reviewers for the thoughtful review. We have considered each comment carefully and have made the changes noted below, which we believe have again improved the manuscript. We have enclosed two versions of the revised manuscript in Word (.docx)—one with changes highlighted under “track changes” and a second “clean” version with all changes accepted.

Please find here our point-by-point responses to reviewer comments in green font, organized by headings taken from the reviewers provided on 19 February 2024 (also, please note that for ease of reference the lines below refer to the “clean” version of the revised manuscript):

*Begin itemized review response.*

**Editor(s)' Comments to Author (if any):**

Journal Requirements:

When submitting your revision, we need you to address these additional requirements.

1. Please ensure that your manuscript meets PLOS ONE's style requirements, including those for file naming. The PLOS ONE style templates can be found at

<https://journals.plos.org/plosone/s/file?id=wjVg/PLOSOne_formatting_sample_main_body.pdf> and

<https://journals.plos.org/plosone/s/file?id=ba62/PLOSOne_formatting_sample_title_authors_affiliations.pdf>

2. Please provide additional details regarding participant consent. In the ethics statement in the Methods and online submission information, please ensure that you have specified (1) whether consent was informed and (2) what type you obtained (for instance, written or verbal, and if verbal, how it was documented and witnessed). If your study included minors, state whether you obtained consent from parents or guardians. If the need for consent was waived by the ethics committee, please include this information.

If you are reporting a retrospective study of medical records or archived samples, please ensure that you have discussed whether all data were fully anonymized before you accessed them and/or whether the IRB or ethics committee waived the requirement for informed consent. If patients provided informed written consent to have data from their medical records used in research, please include this information.

3. Thank you for stating the following financial disclosure:

"This study was supported by the Centers for Disease Control and Prevention, Enhanced Epidemiology and Laboratory Capacity (ELC) grant number: 5-NU50CK000539."

Please state what role the funders took in the study. If the funders had no role, please state: ""The funders had no role in study design, data collection and analysis, decision to publish, or preparation of the manuscript.""

If this statement is not correct you must amend it as needed.

Please include this amended Role of Funder statement in your cover letter; we will change the online submission form on your behalf.

Author response: The following statement is correct "The funders had no role in study design, data collection and analysis, decision to publish, or preparation of the manuscript." Many thanks for seeing to the amendment.

4. Thank you for stating the following in your Competing Interests section: "NA"

Please complete your Competing Interests on the online submission form to state any Competing Interests. If you have no competing interests, please state ""The authors have declared that no competing interests exist."", as detailed online in our guide for authors at http://journals.plos.org/plosone/s/submit-now

This information should be included in your cover letter; we will change the online submission form on your behalf.

Author response: Thank you for offering to change the submission form on our behalf. Co-author J.A.L. discloses the following financial interests/personal relationships which may be considered as potential competing interests: receipt of grants and honoraria from Pfizer, Inc, outside the submitted work. All other authors have declared that no competing interests exist.

5. In the online submission form, you indicated that "De-identified data may be made available upon to the corresponding author with approval from the California Department of Public Health Office of Human Subjects Protection."

All PLOS journals now require all data underlying the findings described in their manuscript to be freely available to other researchers, either 1. In a public repository, 2. Within the manuscript itself, or 3. Uploaded as supplementary information.

This policy applies to all data except where public deposition would breach compliance with the protocol approved by your research ethics board. If your data cannot be made publicly available for ethical or legal reasons (e.g., public availability would compromise patient privacy), please explain your reasons on resubmission and your exemption request will be escalated for approval.

Author response: We have uploaded the deidentified data to Data Dryad for public access and provided the link in the manuscript under “Data Availability” subsection.

6. One of the noted authors is a group or consortium [California COVID-19 Case-Control Study Team]. In addition to naming the author group, please list the individual authors and affiliations within this group in the acknowledgments section of your manuscript. Please also indicate clearly a lead author for this group along with a contact email address.’

Author response: Thank you for this note, we have added authors in that group to the acknowledgements section.

7. Your ethics statement should only appear in the Methods section of your manuscript. If your ethics statement is written in any section besides the Methods, please move it to the Methods section and delete it from any other section. Please ensure that your ethics statement is included in your manuscript, as the ethics statement entered into the online submission form will not be published alongside your manuscript.

Author response: Thank you, we have revised the manuscript accordingly.

8. Please include captions for your Supporting Information files at the end of your manuscript, and update any in-text citations to match accordingly. Please see our Supporting Information guidelines for more information: <http://journals.plos.org/plosone/s/supporting-information>.

Author response: Thank you for this guidance. We have amended in text citations and added captions for the Supporting Information files at the end of the manuscript.

9. Please review your reference list to ensure that it is complete and correct. If you have cited papers that have been retracted, please include the rationale for doing so in the manuscript text, or remove these references and replace them with relevant current references. Any changes to the reference list should be mentioned in the rebuttal letter that accompanies your revised manuscript. If you need to cite a retracted article, indicate the article’s retracted status in the References list and also include a citation and full reference for the retraction notice.

Author response: Thank you, we have reviewed references and adjusted citations per guidance.

Additional Editor Comments:

This study is very interesting, and no major issues were found. Please consider the reviewer comments below, and any changes for improvement will be appreciated.

Author response: many thanks for sharing your thoughts, we appreciate the positive feedback.

Reviewers' comments:

Reviewer's Responses to Questions

Comments to the Author

1. Is the manuscript technically sound, and do the data support the conclusions?

The manuscript must describe a technically sound piece of scientific research with data that supports the conclusions. Experiments must have been conducted rigorously, with appropriate controls, replication, and sample sizes. The conclusions must be drawn appropriately based on the data presented.

Reviewer #1: Yes

2. Has the statistical analysis been performed appropriately and rigorously?

Reviewer #1: Yes

3. Have the authors made all data underlying the findings in their manuscript fully available?

The PLOS Data policy requires authors to make all data underlying the findings described in their manuscript fully available without restriction, with rare exception (please refer to the Data Availability Statement in the manuscript PDF file). The data should be provided as part of the manuscript or its supporting information, or deposited to a public repository. For example, in addition to summary statistics, the data points behind means, medians and variance measures should be available. If there are restrictions on publicly sharing data—e.g. participant privacy or use of data from a third party—those must be specified.

Reviewer #1: Yes

4. Is the manuscript presented in an intelligible fashion and written in standard English?

PLOS ONE does not copyedit accepted manuscripts, so the language in submitted articles must be clear, correct, and unambiguous. Any typographical or grammatical errors should be corrected at revision, so please note any specific errors here.

Reviewer #1: Yes

5. Review Comments to the Author

Please use the space provided to explain your answers to the questions above. You may also include additional comments for the author, including concerns about dual publication, research ethics, or publication ethics. (Please upload your review as an attachment if it exceeds 20,000 characters)

Reviewer #1: In their manuscript, Fukui and colleagues implemented a test-negative case-control study including participants residing in California to inform the design and implementation strategies of observational research in pandemic settings. Results showed that patients with COVID-19 had a higher odds of answering the phone and consenting to participate in the study, compared to control subjects (COVID-19-negative participants). Also, calls placed from 4pm to 6pm had the highest adjusted odds of being answered. The authors also highlighted aspects of interacting with participants with physical and emotional needs, and documented verbal harassment cases from individuals called.

Although the topic may not currently be up-to-date, the study is appropriately designed and well-written. Consider the following for improvement:

1. Lines 1-3: The manuscript mainly focused on identifying factors that might influence participation in a phone-based research survey. The candidate pool was based on a survey examining risk factors for COVID-19 infection. However, the way the title is phrased implies that risk factors for COVID-19 were actively researched in this paper. Please, revise the title to accurately describe the purpose of the study.

Author response: We have revised the title to better reflect this manuscript. It is now titled “Mixed methods approach to examining the implementation experience of a phone-based survey for a SARS-CoV-2 test-negative case-control study in California”.

2. Lines 109-110: Based on supplementary figures S2 and S3, a laboratory test was used for determining eligibility for the study. Why is clinical diagnosis of COVID-19 described as an eligibility criterion in the Methods? Were eligible participants recruited based on lab results, clinical symptomatology, or both?

Author response: The potential participant’s most recent SARS-CoV-2 laboratory test was obtained from case and control linelists generated from all mandatory reporting to the state surveillance system (CalREDIE) within the past 72 hours. Once a case or a control that was matched to an enrolled case on the linelist was called, they were asked this additional question about prior COVID-19 diagnosis or prior positive test results (not including their most recent test that we generating the line lists from). We wanted to ensure that participants, both cases and controls, had no prior infection of COVID-19 before enrolling them in the study.

3. The results showed that individuals with COVID-19 had a higher odds of answering the phone and consenting to participate in the study. From the supplementary materials, it seems that participants were asked to comment on their symptoms, if any. Did the authors run a separate analysis comparing patients with COVID-19 who were asymptomatic vs. symptomatic? If no, could such an approach be explored as well? It might be just a speculation, but I think that symptomatic patients would be more willing to participate.

Author response: Thank you for this suggestion. We did not run this analysis however, we acknowledge the limitation that patients with milder symptoms may be more willing to participate, while those who were more severely ill might have been unreachable, too unwell to give informed consent, or less willing to participate.

*End itemized review response.*

We would, again, like to thank the editor and reviewers for their time and thoughtful comments. We believe that edits made in response to the reviewer notes have further strengthened the manuscript and we look forward to receiving your feedback in due course.

Kind Regards,

Jake M. Pry, PhD, MPH
Assistant Professor
Department of Public Health Sciences, University of California, Davis
jmpry@ucdavis.edu | jake.pry@cidrz.org
